# A structured elicitation method to identify key direct risk factors for the management of natural resources

**DOI:** 10.1016/j.heliyon.2015.e00043

**Published:** 2015-11-24

**Authors:** Michael Smith, Ken Wallace, Loretta Lewis, Christian Wagner

**Affiliations:** a Science Division, Department of Parks and Wildlife, 17 Dick Perry Avenue, Technology Park, Western Precinct, Kensington, WA 6151; b School of Agricultural and Resource Economics, the University of Western Australia, 35 Stirling Hwy, Crawley, WA 6009, Australia; c Horizon Digital Economy Institute & IMA Group, School of Computer Science, University of Nottingham, Nottingham, United Kingdom; d Australian Wildlife Conservancy, PO Box 8070 Subiaco East, WA 6008, Australia

**Keywords:** Ecology, Risk management, Decision analysis, Biological sciences

## Abstract

The high level of uncertainty inherent in natural resource management requires planners to apply comprehensive risk analyses, often in situations where there are few resources. In this paper, we demonstrate a broadly applicable, novel and structured elicitation approach to identify important direct risk factors. This new approach combines expert calibration and fuzzy based mathematics to capture and aggregate subjective expert estimates of the likelihood that a set of direct risk factors will cause management failure. A specific case study is used to demonstrate the approach; however, the described methods are widely applicable in risk analysis. For the case study, the management target was to retain all species that characterise a set of natural biological elements. The analysis was bounded by the spatial distribution of the biological elements under consideration and a 20-year time frame. Fourteen biological elements were expected to be at risk. Eleven important direct risk factors were identified that related to surrounding land use practices, climate change, problem species (e.g., feral predators), fire and hydrological change. In terms of their overall influence, the two most important risk factors were salinisation and a lack of water which together pose a considerable threat to the survival of nine biological elements. The described approach successfully overcame two concerns arising from previous risk analysis work: (1) the lack of an intuitive, yet comprehensive scoring method enabling the detection and clarification of expert agreement and associated levels of uncertainty; and (2) the ease with which results can be interpreted and communicated while preserving a rich level of detail essential for informed decision making.

## 1 Introduction

World-wide, natural resource managers often struggle to achieve operational goals given the combined impacts of: (1) our frequently poor understanding of the complexities of nature (Cilliers et al., 2013), (2) the plethora of processes and associated risk factors that require management (e.g., Dudgeon et al., 2006, Salafsky et al., 2008) and (3) resource limitations (Tulloch et al., 2015). To address these issues, managers must assess the likelihood of meeting management targets, particularly in the face of numerous direct risk factors (Metcalf and Wallace 2013). For this work, direct risk factors are those that directly affect the capacity of biological elements (see definition in Table 1) to survive and reproduce at a sufficient rate to maintain populations (e.g., Metcalf and Wallace 2013). Risk analysis is an important component of decision making that, combined with other socio-economic data, contributes to selecting priority management actions (Gregory et al., 2012). In general, it would be pointless to invest resources in actions where failure to meet management targets is likely (Joseph et al., 2009).

**Fig. 1:**
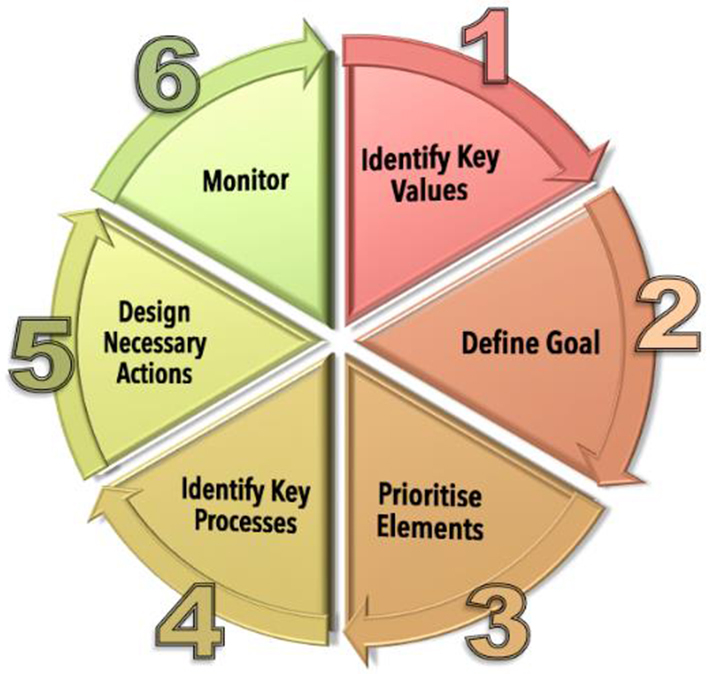
Diagram of general management approach (as described by Wallace 2012) within which the risk factor analysis sits. The direct risk factor analysis fits in step 4 and is used to ultimately identify important processes for management.

Nevertheless, as emphasised by Gregory et al. (2012), it is important to view risk analysis in the wider context of management. Specifically, it is one component of a much more complex array of planning and decision-making tools that need to be applied, and ultimately it is human values that drive the choices that are necessarily made during environmental decision making (Gregory et al., 2012, Wallace, 2012). Here, we are following the planning framework described in detail by Wallace (2012), which is outlined in Fig. 1. Thus, it is important to emphasise that the work described in this paper is only one component of a much larger body of work that involves on-going engagement with stakeholders, including local landholders (Wallace et al., 2011) and experts. For example, the priority values used as a basis for setting the management goal and targets were selected by stakeholders (Wallace et al. unpublished data).

Effective risk analysis in operational, natural resource management depends on establishing and applying:

a. Clear management goals, preferably couched in terms of priority human value(s) (defined in Table 1) and thus wellbeing (Wallace 2012), but at the least meeting criteria for a fundamental objective as described by Gregory et al. (2012). Such outcome-based goals connect decisions with human values and provide a sound basis for generating management targets for risk analyses that ultimately assess, together with appropriate measures of uncertainty, the probability of achieving operational goals and targets.
b. Specific temporal and spatial scales over which the management goal and targets are to be achieved (USEPA, 1998, Wallace, 2006, Gilioli et al., 2014). For example, whether the impacts of a direct risk factor, such as an environmental weed, are to be assessed over one week or 30 years, or over 10 square km or 10,000 square km, profoundly affects the outputs from risk analyses.
c. A classification of direct risk factors that forms a coherent set suitable for analysis (Wallace, 2012, Metcalf and Wallace, 2013). At a minimum, classifications of risk factors should: (1) be comprehensive for the specific task; (2) minimise redundancy, as this leads to double counting and linguistic uncertainty; and (3) be constructed from comparable entities to minimise category errors. For example, in relation to the last point, risk factors should not be a mix of system processes, system properties and system elements (see definitions of terms in Table 1), but should comprise only one of these entities counted at a consistent point in any causal chain (Wallace, 2012, Metcalf and Wallace, 2013).
d. Suitable methodologies for capturing and analysing uncertain data for risk analyses. Given the significant gaps in knowledge and lack of management resources outlined above, and the frequent need to make decisions despite these constraints, elicitation of estimates from experts is commonly used in risk analyses, although such approaches require careful management to avoid a wide range of problems such as linguistic uncertainty, expert bias, and halo effects (Burgman, 2005, ACERA, 2010, Donlan et al., 2010, Gregory et al., 2012, Martin et al., 2012).
e. Methods for documenting and communicating (a) to (d) above that are transparent, readily communicable and provide a firm basis for continuing adaptation and amendment. This is particularly important in natural resource planning and operations where communication with a wide range of stakeholders from highly varied backgrounds is frequently required, and clarifying trade-offs and synergies amongst competing interests is critical to effective planning. Given the significant difficulty most people have accurately interpreting probabilistic data (Kahneman 2011), it is critical that the analysis and presentation of risk analysis outputs is unambiguous with uncertainties clearly identified.

Finally, all the above must be implemented in a way that is sufficiently cost-efficient so that community groups, and managers with limited resources, can implement the techniques. Although there are a wide range of one-on-one and group techniques for eliciting information from experts (Burgman, 2005, Gregory et al., 2012, Martin et al., 2012), we continued with the earlier approach outlined in Metcalf and Wallace (2013), which is a group elicitation method involving discussion, but within which scoring is anonymous and the experts are calibrated. Given the limitations of time and resources, and the success of the prior work, for the case study described here this was deemed the most appropriate methodology with the adjustments outlined below.

In earlier applied planning by Metcalf and Wallace (2013), the focus was on implementing an expert elicitation approach ACERA, 2010, Speirs-Bridge et al., 2010) with an emphasis on avoiding linguistic uncertainty. Although this entailed addressing each of (a) to (e) above, a weakness identified during the study was that the scoring approach was not intuitive and was difficult to explain where access to expertise was highly time-limited. Also, on later reflection it was considered that the uncertainty attached to expert responses, and the level of agreement amongst them, should be more overtly captured, documented and displayed in a more readily communicated format. This was particularly important given that managers are working with multi-stakeholder advisory groups. Finally, the original work only dealt with risk factors associated with hydrological processes and it was important to test the approach with a more diverse group of risk factors.

Consequently, the aim of the work reported here was to build on the elicitation approach used by Metcalf and Wallace (2013) by:

i. Improving the analytical and communication aspects of the earlier work. This was largely achieved through incorporating the ‘ellipse’ based interval agreement approach of Wagner et al., (2014), which is described in detail in the methods section below.
ii. Incorporating mathematical analysis of the expert responses (and associated uncertainty), providing easily interpretable models of expert-group levels of agreement and overall uncertainty.
iii. Treating a full range of risk factors, rather than those restricted to hydrological processes.

As with the original work, the methodologies were developed and tested in actual applied planning, in this case the development of a management plan for the Lake Bryde Catchment in south-western Australia. The case study is described in a step-by-step manner so that potential practitioners will be more able to adapt the approach to suit their management situation. We consider this to be particularly important, because experience has taught us that no matter how novel, interesting or broadly applicable an approach, its adoption by practitioners depends on the methods being readily applied with immediate operational benefits.

## 2 Methods

The case study planning exercise was for the management of important biological elements (Supplementary material 1) in the Lake Bryde Natural Diversity Recovery Catchment (the catchment) in Western Australia (Walshe et al., 2004). The broader planning approach that this work fits within is characterised in Fig. 1. The risk analysis described in this paper is ultimately used to identify important processes for management (Step 4 in Fig. 1). This management framework is described in detail in Wallace (2012).

A workshop was run by a planning project team to identify important direct risk factors for the set of biological elements. The biological element list (Supplementary material 1) was developed through a separate process using catchment stakeholder representatives in a previous workshop who were assisted by the project team and by technical experts (Wallace et al. unpublished data). The stakeholder group included representatives of key groups in the area including land owners, education, tourism, agriculture, water management, etc. (Wallace et al. unpublished data).

### 2.1 The experts

To identify key risk factors, a group of experts were invited to attend a meeting held on November 25th 2014 to assess the level of risk to the biological elements of the catchment. The experts had different education, training and experience, but were all, through their work, familiar with the biological elements and the various risk factors. Specifically, individuals were assessed as being suitable experts against six criteria: (1) relevant science experience/knowledge (formal and/or practical experience; critical), (2) natural resource management knowledge/experience in the relevant biogeographic region of Western Australia (critical), (3) a general understanding of the potential risk factors (critical), (4) detailed understanding of a subset of the risk factors (critical), (5) natural resource management knowledge/experience in the Lake Bryde catchment (preferable) and (6) a knowledge/understanding of the planning approach (preferable). Apart from one expert (who was a local land owner), all were drawn from within the Department of Parks and Wildlife (the department). However, those from within the department represented different divisions and branches (e.g., operations, research, planning) and half of the expert group worked in the catchment area and as a result had close working relationships with the landowners that surround the wetland complex and its biological elements. As such, the experts as a group had a high level of understanding of local issues.

### 2.2 The workshop

#### 2.2.1 Expert calibration

To manage over-confidence, which is a common issue in expert elicitation exercises, the opinions of each expert were weighted by their ability to answer a series of calibration questions (Burgman, 2005, Speirs-Bridge et al., 2010, Metcalf and Wallace, 2013). During the workshop, but before the individual elicitation exercise, each expert was asked to anonymously answer a series of questions (Supplementary material 1) that were relevant to the management issues and for which experts were unlikely to precisely know the correct answer (based on best available information). The experts answered the questions using the same interval-valued questionnaire approach used for the actual risk analysis described below and thus the calibration exercise also provided training for the risk analysis. Each expert was asked to:

1) draw an ellipse, the ends of which represented their lowest and highest estimates for the correct response to each question along a scale ranging from zero to one.
2) draw a point (or cross) within each ellipse to indicate their best estimate.
3) look at the ellipse and assess their level of confidence (50 to 100%) that the true answer lay within the ellipse.

#### 2.2.2 Calibration analysis

Based on available knowledge, the most likely answer to each calibration question (Supplementary material 1) was initially determined by the planning project team. After the workshop, the answers were discussed amongst the experts via email to ensure the ‘correct’ answers were acceptable to the experts. To make the expert ellipses comparable we used simple linear extrapolation (e.g., Speirs-Bridge et al., 2010) of the expert's best estimate and confidence that the ellipse captured the true value to adjust their initial interval to create a derived 80% confidence interval (CI; Supplementary material 2). Note, derived 80% CIs were chosen to facilitate comparison with other studies (e.g., Speirs-Bridge et al., 2010, Metcalf and Wallace, 2013). However, the CI could be adjusted to any confidence level deemed appropriate to the situation. The proportion of the expert's total derived 80% CI that overlapped with the pre-determined interval for a correct answer was calculated. To calibrate each expert, their correct proportions were averaged. The calibration scores were used to weight the elicitation scores in the aggregation process described below (refer to Supplementary material 1) to reduce the effects of over-confidence.

#### 2.2.3 The biological elements

In the workshop, the biological elements were presented to the expert group with a description of the component species that characterise each element and a map of each element's spatial distribution – as defined during a previous workshop. The biological element list is provided in Supplementary material 1.

#### 2.2.4 Classifying the direct risk factors and identification of the most susceptible species

A direct risk factor list similar to the one used by Metcalf and Wallace (2013), but not focused solely on the management of hydrology, was developed by the experts (Table 2). To help the experts estimate likelihoods of management target failure, the species in each element thought to be most susceptible to the direct risk factors were identified by the group. A comprehensive discussion was encouraged to bring the experts to a similar level of understanding in terms of the kinds of species that are most likely to be affected by the different risk factors over the management period.

#### 2.2.5 Current management

The group discussed existing management practices in the catchment so that all experts had a comparable understanding of current management. In general, existing management at the time focused on problem species (i.e. weed, rabbit, fox and kangaroo control), revegetation and maintenance of water management infrastructure (i.e. waterways, bores, etc.). It was also noted that non-departmental management must be considered, including farm practices (i.e. revegetation, pest species control, private water management such as draining and damming water, etc).

#### 2.2.6 Management target

After a group discussion among the experts within the workshop, a single ‘element level’ management target was defined as:

*With current management, no loss of the natural species that characterise the element over the 20 year management period.*

All risk assessments were estimated against this target for each element. Below, the phrase ‘management target failure’ means failure to achieve the above target.

#### 2.2.7 Initial group elicitation

To reduce the number of element-risk factor combinations to be assessed in detail (total number = 322; 23 risk factors and 14 elements), the experts were initially asked to work as a group to identify any risk-element combinations that were thought to present a 5 % or less chance of causing management target failure over the management period (e.g., Metcalf and Wallace 2013). These combinations were removed from the more detailed assessment, and by doing so, the group removed 297 risk factor-element combinations, leaving 53 to be assessed in detail (Supplementary material 3).

#### 2.2.8 Expert elicitation

As with the calibration exercise, to elicit expert opinions, the methods of Wagner et al. (2014) and the 4-step elicitation approach of Speirs-Bridge et al. (2010) and Metcalf and Wallace (2013) were combined to elicit and aggregate the opinions of the experts individually and anonymously. Specifically, for each of the 53 risk factor-element combinations considered, the experts conducted an assessment of the likelihood of management target failure using the same ellipse approach described for the calibration exercise.

The location of the ellipse on the scale is the expert's estimate of the likelihood that the risk factor will cause the loss of any one or more natural species that characterise an element over the management period, and the width of the ellipse captured their uncertainty about this likelihood (i.e., highest and lowest estimates as represented by the end points of the ellipse). Finally, because experts are thought to be better at judging intervals than they are at creating them (Speirs-Bridge et al., 2010), each expert was asked to look at the ellipse they had created and to assess their level of confidence that the true likelihood lay within the ellipse. The workshop facilitator talked through a hypothetical example with the group and during this presentation any issues concerning the elicitation methods were addressed.

#### 2.2.9 Identifying the most important risk factors

To combine the expert opinions, the ellipses (which are used to generate intervals: lowest and highest points of the ellipse), which encode the expert beliefs were aggregated across all experts based on the interval agreement approach of Wagner et al. (2014). The interval agreement approach creates a distribution, specifically a fuzzy set from intervals contributed by different sources – in our case, from different experts. In order to create this distribution, the interval agreement approach weights parts of intervals which are in agreement with parts of intervals from other experts more highly, thus extracting agreement over all contributed intervals. Based upon the ‘raw’ estimates, each overlapping interval (one from each expert) will contribute a ‘1’ to the overall score and so complete agreement will score ‘10’ as there were ten experts. To correct for the calibration results, the expert contribution will be ‘1’ if the expert is perfectly calibrated or less than ‘1’ where the expert is not perfectly calibrated (Supplementary material 1).

**Fig. 2:**
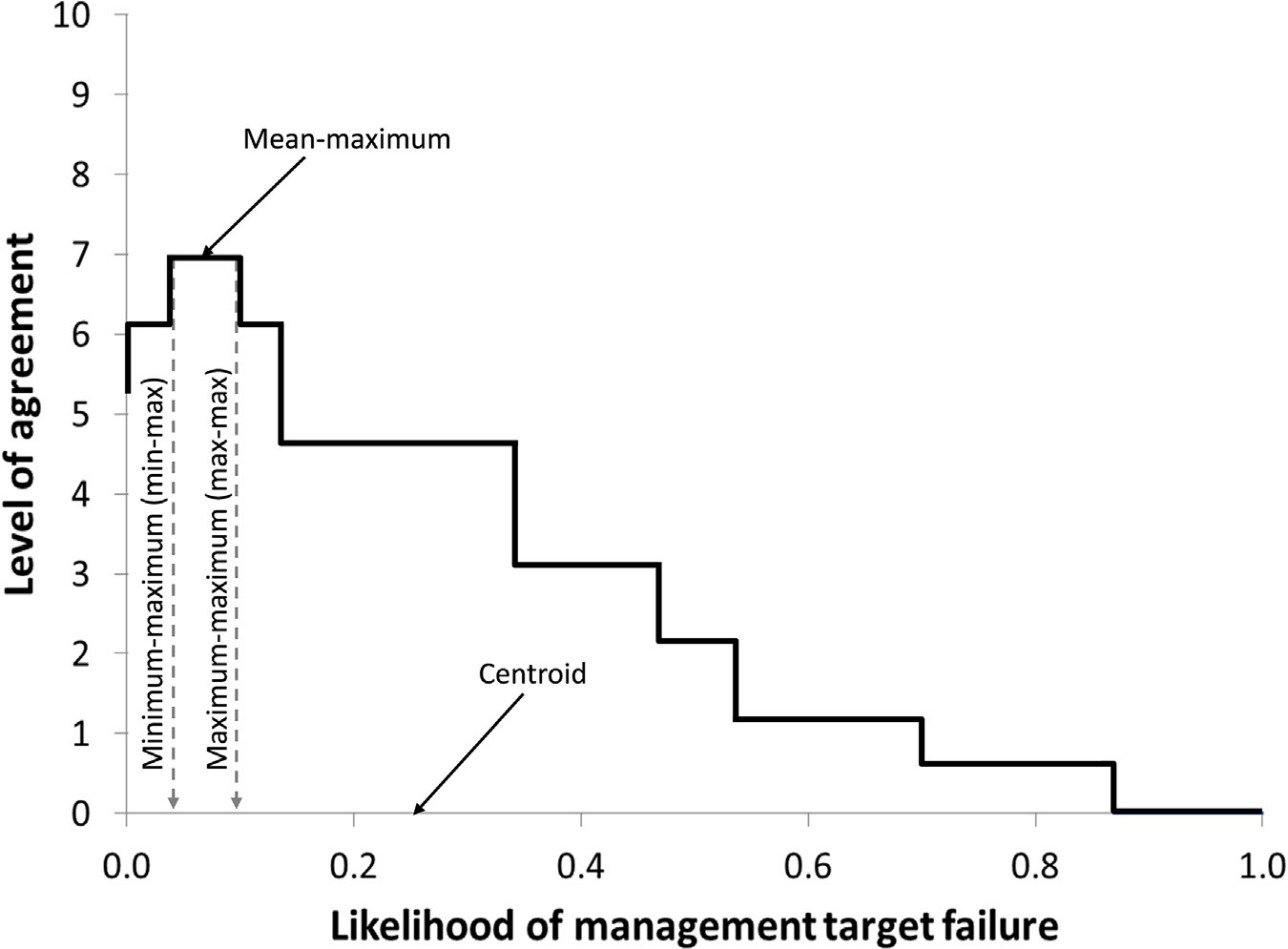
Explanation of some of the methods that can be used to extract a crisp likelihood value (from the overall, expert-group based distribution) that a management target will not be met over the 20 year period.

For each risk factor-element combination, each ‘raw’ interval (as captured by the ellipse) was first aggregated (the Raw-estimates; Supplementary material 1, 2 and 3). An example of the aggregation process is provided in Supplementary material 2 (in Microsoft Excel 2010™). Each initial ellipse was then adjusted to 80% derived CI using simple linear extrapolation and these estimates were aggregated (CI-estimates; Supplementary material 1, 2 and 3). The CI-estimate intervals were then adjusted for the expert's calibration scores and these estimates were aggregated (Calibrated-estimates; Supplementary material 1, 2 and 3). One could simply generate the Calibrated-estimates, but we find it useful to compare the different steps to assess changes from raw to fully calibrated and adjusted estimates. For each risk factor-element a min-max score was estimated from the aggregated data. The min-max score is the minimum score along the x-axis that corresponds with the highest level of agreement (Fig. 2). This is one estimate of the likelihood that the risk factor will cause target failure. Other estimates could be used, such as the centroid (the centre of gravity in terms of the area under the curve which can be thought of as representing the point upon which the graph will balance; Fig. 2, also see Mendel 2000), max-max (maximum score along the x-axis that corresponds with the highest level of agreement), and so on (Fig. 2). In discussion with the expert group we chose to use the min-max estimate which provides a lower score at which target failure will occur when compared to the centroid or max-max estimates. In other scenarios practitioners may choose to use an alternate metric, such as the centroid or max-max estimates. Either way, it will be appropriate to discuss the choice of metric with experts and/or stakeholders.

## 3 Results

### 3.1 Correcting for confidence and the expert calibration

The experts differed in their overall calibration weighting (Mean = 0.40, Range = 0.25 to 0.50). The reasons for this overall weighting are discussed below. Aggregation graphs for each calibration question are provided in Supplementary material 1. From these graphs, we can see that the experts were, in general, in agreement on the answers (e.g., examine the graph for question 1, which shows a high level of agreement). However, one question in particular, relating to the richness of the mallee vegetation community (question 4) evoked a strongly bimodal response, indicating that the group was split on their opinions. Interestingly, the group was in high agreement that *Banksia xylothemelia* is an obligate seeder (question 2) even though it is not (Supplementary material 1). Overall, we considered the accuracy of the experts to be satisfactory, although the results underline that there are divergences of views and that there are certainly some areas of inadequate knowledge. That all results are presented together to stakeholders allows independent assessment of whether the experts are sufficiently accurate from the perspective of each stakeholder. It should be noted that there is no ‘perfectly acceptable’ level of accuracy. The reality is that one uses the best experts that are available under the circumstances, and we consider that the level of accuracy generated by the case study experts is sufficient to ensure that, as a group, they provided a much better analysis of risk than any one of the experts taken individually, or by basing assessment of risk on intuitive experience and undocumented rules of thumb.

### 3.2 Risk factor analysis

**Fig. 3:**
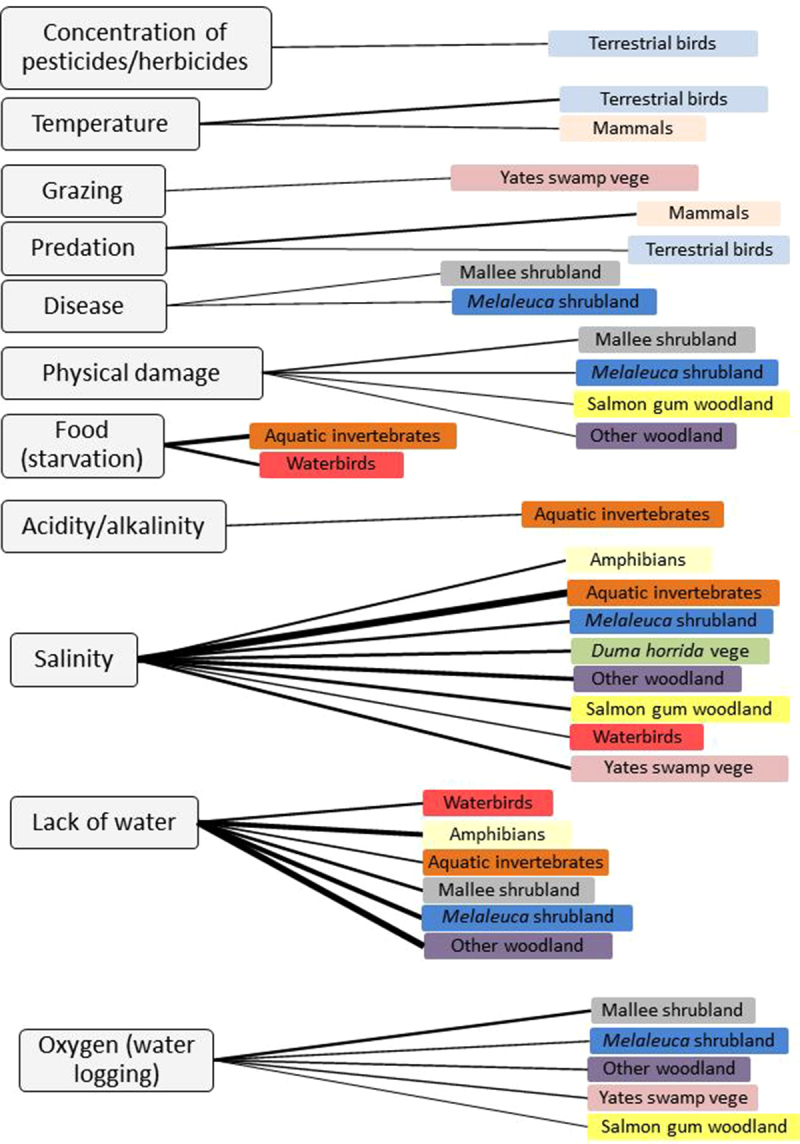
Characterisation of the likelihood that a direct risk factor (light grey box) will cause management target failure for each affected biological element. The estimated likelihood of species loss (min-max – described in the main text) over the 20-year management period for each risk factor-element combination is expressed by the thickness of the black line (thicker the line, the greater the likelihood of management target failure) between each risk factor and the elements. Actual likelihoods are provided in Supplementary material 3. Risk factor-element combinations with a likelihood of 5% or less of causing management target failure are not shown.

In general, the experts showed strong agreement in their beliefs about the likelihood that each risk factor will cause the loss of natural species over the management period (some example aggregation graphs are provided in Supplementary material 3). The Raw-estimates, CI-estimates and Calibrated-estimates for each risk factor-element combination are also provided in Supplementary material 3. From Fig. 3 (and Supplementary material 3), we can see that, in terms of the number of affected elements, salinity was believed to be a particularly important risk factor for the most elements (n = 8) followed by a lack of water (drought; n = 5 and inappropriate hydro period; n = 1) and lack of oxygen (waterlogging; n = 5), physical damage (fire and other disturbances; n = 4), lack of food (n = 2), predation (n = 2), temperature (n = 2), disease (n = 2), acidity/alkalinity (n = 1), grazing (n = 1) and pesticides/herbicides (n = 1).

In terms of the actual likelihood that a particular risk factor will cause management target failure, salinity (aquatic invertebrates, other woodlands and Salmon gum woodland), lack of water (other woodlands, amphibians, *Melaleuca* shrubland and, mallee shrubland) and lack of food (aquatic invertebrates and waterbirds) were the most threatening (Fig. 3; also refer to Supplementary material 3).

## 4 Discussion

In this paper we demonstrate a novel and broadly applicable approach to assess the level of threat to important elements of natural systems by combining the risk analysis approach of Metcalf and Wallace (2013) with the interval agreement approach of Wagner et al. (2014). The approach has broad application in the area of risk assessment, but can also be applied in any area where expert opinion (and associated uncertainty) requires capture, aggregation and communication. Smith et al. (unpublished data), Wallace et al. (unpublished data) and Wagner et al. (2014) provide examples of how the interval agreement approach may be used in a variety of situations. For the case study, opinions elicited from experts identified a number of factors that are considered to pose a significant risk to the priority biological elements of the Lake Bryde catchment. The results suggest that, without additional management to that currently occurring, there is a high likelihood that natural species will be lost from the elements, constituting management target failure. Because the management target was set around a property (species richness) that directly influences element value (Smith et al. unpublished data), target failure will translate into goal failure for the catchment which is to “*maintain or improve the knowledge-heritage, recreation and future option values provided by the catchment's priority biological elements for the next 20 years*” (based on the most important values elicited from stakeholders; Wallace et al. unpublished data).

Importantly, the approach provides a pathway to garner essential information for prioritizing elements for future management attention. Developing a detailed understanding of the processes that control important risk factors is the next important planning step. From that analysis, a series of management activities can be developed and scrutinised (Step 5; Fig. 1) in terms of their effectiveness (Hockings et al., 2006), feasibility (Groves et al., 2002) and cost (Joseph et al., 2009) and managers can set more realistic management targets and/or allocate additional resources to manage specific risk factors directly. For the case study, these planning steps are all undertaken in conjunction with stakeholder and expert advisory groups, which includes catchment landholders.

Two important benefits of the approach are that it can be enacted by people with minimal statistical expertise, and the generation of the expert opinion aggregation graphs in workshops provides an excellent communication tool. Here, the employed interval agreement approach, which avoids assumptions such as outlier removal or model fitting (e.g., to normal distributions), substantially support interpretability and transparency of the data analysis. In addition to allowing a range of statistics to be generated to express the levels of risk and the associated uncertainties, the graphs provide a powerful visual interpretation of the expert opinions including their level of agreement, an important aspect of expert elicitation (Gregory et al., 2012). Thus, the graphs help to explain the results and to generate dialogue and additional input from the experts. In particular, the approach not only allows the rating of risks, but also an assessment of the level of agreement amongst experts concerning these ratings. The authors have now successfully used this general approach in a number of different applications, such as rating the relative importance of human values arising from a given set of elements (Wallace et al. unpublished data) and rating elements on their importance for deriving human values (Smith et al. unpublished data). In all cases we have had very positive feedback from the associated participants, which underlines that the approach facilitates dialogue and improves understanding and communication amongst participants.

For the case study, in terms of the broader processes that frame each key risk factor, we initially suggest that understanding the issues that relate to hydrology (*salinity*, *lack of water*, *water logging*, *acidity/alkalinity* and *food availability*) will be particularly important. Issues relating to problem species (*predation*, *grazing*, and *disease*), farm management (*pesticides/herbicides*), fire (*physical damage*) and climate change (*temperature*) will also warrant consideration, while bearing in mind that at least some of the risk processes will themselves interact with each other. Managing all of these factors and their interactions is likely to be extremely challenging and will demand substantial resources, highlighting the need to establish clear priorities amongst both the elements and the suite of available management actions. For example, determining how the catchment's hydrology influences *food availability*, *salinity*, *acidity/alkalinity*, *water availability* and *periodicity*, and to then devise justifiable, effective and feasible management activities within the context of issues such as surrounding land use and climate change, are the next major planning challenge for the case study. These complexities of risk factors are not atypical in the management of natural resources (e.g., Hester and Hobbs, 1992, Fitzpatrick et al., 2008, Sherwin et al., 2012).

Provided they meet the five criteria for effective risk analysis outlined in the introduction, expert-based approaches such as that outlined above are far preferable to intuitive, poorly documented management decisions. Nevertheless, the frailties of experts do need to be acknowledged. Such frailties include cognitive bias, framing effects influencing outputs, over-confidence, motivational bias, and aspects related to the cultural-philosophical context (Burgman, 2005, Gregory et al., 2012, Martin et al., 2012). Although the methods used in the case study seek to reduce these frailties as far as practicable, it is not possible to remove them entirely. Consequently, it is essential that the impacts of decisions based on expert analyses are monitored and reviewed as new information arises. Importantly, analyses such as that above may be used to better direct research programs towards tackling the most pressing problems facing managers.

In conclusion, consistent with the case study aims, the described approach dealt with a full range of risk factors using a novel data capture and aggregation process that, based on participant and facilitator comments, significantly improves analysis and communication of risk outputs, and allowed a more intuitive approach to scoring. The approach is fully documented, and thus provides a sound basis for critical review and further development. In particular, the proposed approach is unique in explicitly capturing expert uncertainty (at the level of each individual expert), calibrating the resulting expert-specific interval-valued data and, most significantly, providing a pathway to generate a comprehensive yet easily interpretable cross-expert agreement model without relying on assumptions such as outlier removal or model fitting. The latter in particular strengthens interpretability, accountability (i.e. individual expert-input can be identified in models), while the overall model itself enables managers to rapidly assess expert-group agreement (and discord) — thus enabling the delivery of an appropriate planning strategy. In achieving this outcome, the approach has also covered off the five criteria for effective risk analysis outlined in the introduction.

## Declarations

### Author contribution statement

Michael Smith: Conceived and designed the experiments; Performed the experiments; Analyzed and interpreted the data; Wrote the paper.

Ken Wallace: Conceived and designed the experiments; Performed the experiments; Wrote the paper.

Loretta Lewis: Performed the experiments; Analyzed and interpreted the data; Wrote the paper.

Christian Wagner: Conceived and designed the experiments; Analyzed and interpreted the data; Wrote the paper.

### Competing interest statement

The authors declare no conflict of interest.

### Funding statement

This work was supported by the Western Australian Department of Parks and Wildlife as part of the State Salinity Strategy and Natural Diversity Recovery Catchment Program. This work was partially supported by the UK EPSRC (EP/K012479/1), RCUK (EP/G065802/1) and NERC (NE/M008401/1) grants.

### Additional information

No additional information is available for this paper.

## Figures and Tables

**Table 1:** Terminology: All real world systems (including ecosystems) may be viewed as consisting of the following entities.

Term	Definition
Elements	Material (i.e. physical) things, that are generally classified into biotic (biological) elements such as plants, animals, vegetation units; and abiotic elements such as rocks, water, and mountains.
Processes	Processes are the complex interactions (actions, events, reactions or operations) among and within elements that lead to a definite result (adapted from Tirri et al., 1998) at a given point in time. Threatening processes are those processes that put management goals at risk. Given that there is ambiguity concerning the term ‘threatening processes’ (see Wallace 2012), the term risk factor (see below) is used here.
Properties	Properties are terms that describe the elements of a system, or related processes, or the system as a whole (Wallace 2012). Properties include, for example, the hardness, colour or reactivity of elements; the specific rate of processes; or the salinity, resilience, or sustainability of a system.
Risk factors	Risk factors are those factors that reduce the capacity of biological elements (see definition above) to survive and reproduce at a sufficient rate to maintain or increase populations (e.g., Metcalf and Wallace 2013). Direct (or key) risk factors are those, e.g., predation or starvation (insufficient food resources), that are the ultimate cause of a biological element's reduced capacity to survive and reproduce (see Wallace 2012 for a more detailed explanation).
Systems	A unit formed by all the elements (biotic and abiotic) of a defined space and their interactions with each other. That is, a unit consisting of a set of elements and related processes. The term ‘system’ is used rather than ‘ecosystem’ given that the latter term is variously used in the literature, with associated ambiguity concerning its use in a particular context.
Values	The preferred end-states of human existence, including those required for survival and reproductive success, which, taken together encompass human well-being (Wallace 2012). Examples include adequate resources (e.g., food and water), aesthetic pleasure, meaningful occupation, a benign physical and chemical environment, and spiritual-philosophical contentment

**Table 2:** List of direct risk factors used in the expert analysis. Factors marked with an asterisk were taken forward into the more detailed analysis.

Category of factor	Direct risk factor (all expressed as properties of systems, elements or processes)	Implications in the Lake Bryde Wetland complex (generic examples)
Physical and chemical factors	Acidity/alkalinity*	Increased contaminants through catchment run-off into wetlands may cause death of organisms
Concentration of heavy metals	As above
Concentration of hormones	As above
Concentration of nitrogen	As above
Concentration of other toxins	As above
Concentration of pesticides/herbicides*	As above
Concentration of phosphorus	As above
Carbon dioxide concentration	Anoxic conditions in wetlands may ‘suffocate’ organisms
Physical damage (including fire, wind, flood flow – expressed as frequency of force per unit area, or similar measures)*	Destruction of organisms by fire, flood flow, etc.
Salinity*	Rising saline ground waters and increasing salinity of inflows is causing death of organisms
Temperature (expressed as periods of time above or below specified thresholds)*	With increasing temperature extremes, there is increasing potential for deaths in wetland organisms and vegetation.
Resources (all expressed as amount of resource available per population individual per time)	Food (starvation)*	Mortality following waterlogging and death of trees that provide food
Lack of water (dehydration and inappropriate hydroperiod)*	Extended summer droughts may cause dehydration and death or extended periods without flooding, in a drying climate, may cause failure to regenerate
Life media and substrates	Reduced aquatic substrate e.g., for emergence of invertebrates, due to death and decay (without replacement) of woody aquatic plants
Light deficit	Lack of light penetrating water (e.g., due to increased turbidity) may cause photosynthetic failure
Oxygen (water logging) deficit*	Rising water tables and/or unusually wet cyclonic events may drown vegetation
Disease/competition/predation/etc.	Disease, parasites (concentrations of disease organisms/parasites)	Surface inflows transport diseased plants into the system causing plant death
Grazing (expressed as grazing intensity per population units)*	Grazing as a form of predation causing plant death
Predation (expressed as predation intensity per population units)*	Death of birds due to predation following reduced availability of roosting habitat (due to tree deaths)
Toxic species (expressed as frequency of encounters with toxic species)	Death of animal through consumption of toxins
Reproduction	Lack of genetic diversity (expressed as population genetic diversity)	Reduced genetic diversity following death or emigration resulting in lower reproductive success and survival
Lack of mates (senescence) (expressed as probability of encounters with sexually mature/available members of the opposite sex of the same species)*	Reduced availability of mates due to death or emigration
Lack of nesting habitat (expressed as amounts of nesting habitat per unit area)	Reduced availability of nesting habitat due to inundation
