# Supplementary material for: A structured elicitation method to identify key direct risk factors for the management of natural resources
Source: Heliyon. 2015 Nov 24;1(3):e00043. doi: 10.1016/j.heliyon.2015.e00043 (PMC4945618; doi:10.1016/j.heliyon.2015.e00043)
Supplement: Supplementary material 1 [file mmc1.pdf]

Supplementary Material 1: Biological element list, description of the interval agreement approach and expert calibration information.

Biological element list

Table S1.1: The key natural biological elements for the Lake Bryde catchment. These elements were developed by experts in conjunction with stakeholders.

**Biological element**	**Biological element**
Amphibians	Reptiles
Aquatic invertebrates	Salmon gum woodland
Duma horrida vegetation community	Samphire vegetation community
Mallee shrubland	Terrestrial birds
Mammals	Terrestrial invertebrates
Melaleuca shrubland	Waterbirds
Other woodlands	Yate swamp vegetation community

Interval Aggregation

The interval agreement approach of Wagner et al. (2014) creates fuzzy sets by aggregating a set of ellipses. Figure S1.1 provides an explanatory example of the approach. In the example three experts have drawn ellipses. Note, in this example, the ellipses have not been adjusted to a standard confidence interval. Where the full ellipse information is to be used, the areas of overlap are simply summed with each expert receiving a score of ‘1’. Where experts have been calibrated — in this hypothetical case to 1.0, 0.8 and 0.5 — the calibration (or weighting) scores are summed.

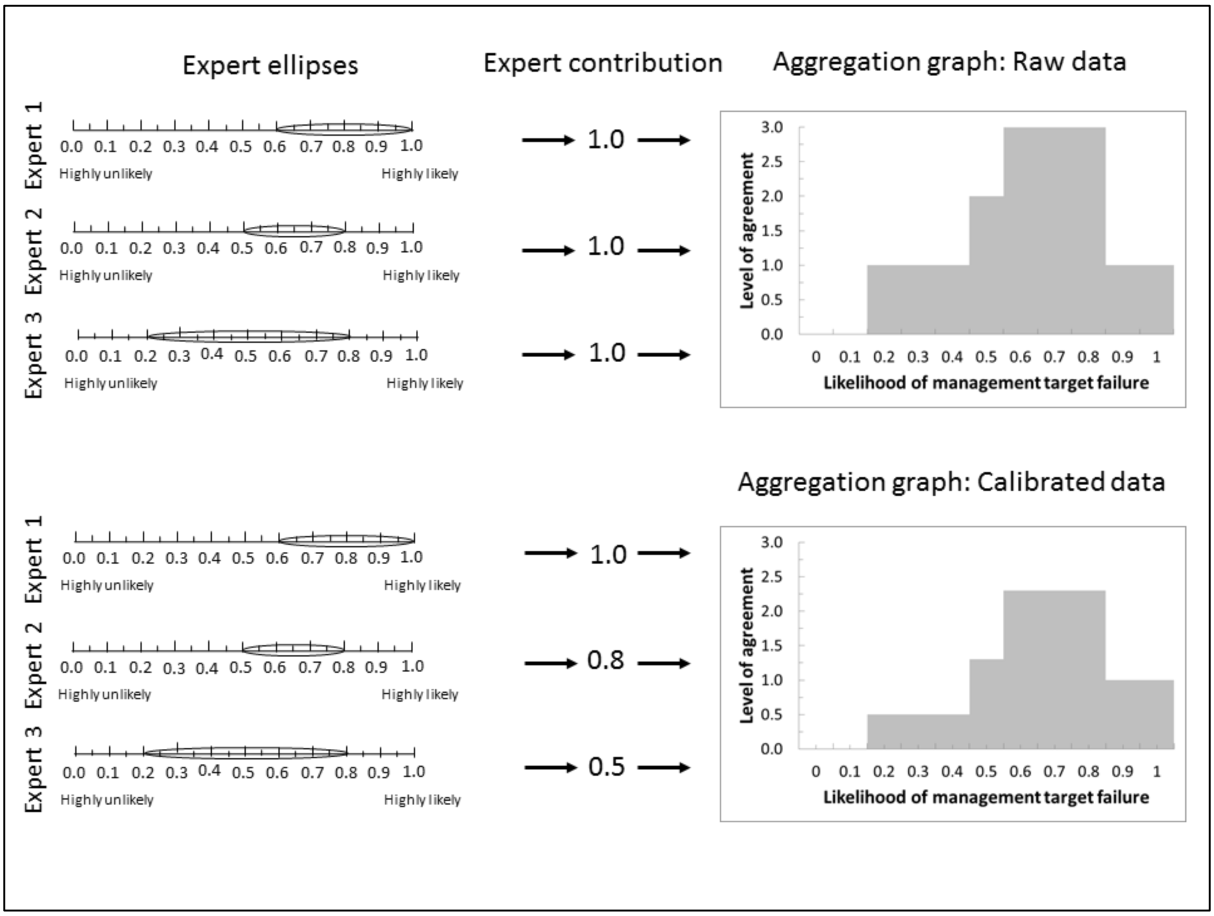

Figure S1.1: Description of the methods used to aggregate data.

Calibration questions, elicitation scale and correct likelihood (grey box).

1) Compared to the other vertebrate elements, how likely is it that the total species richness of the amphibian element is the highest?

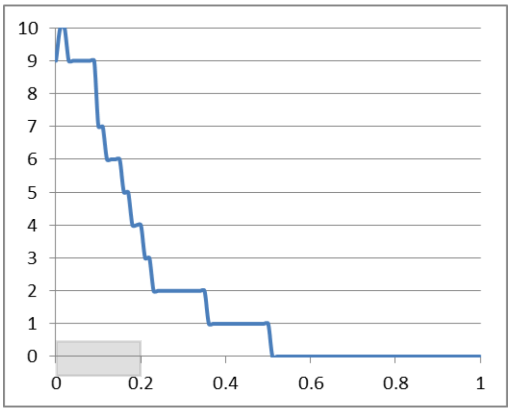

2) How likely is it that *Banksia xylothemelia* is an obligate seeder?

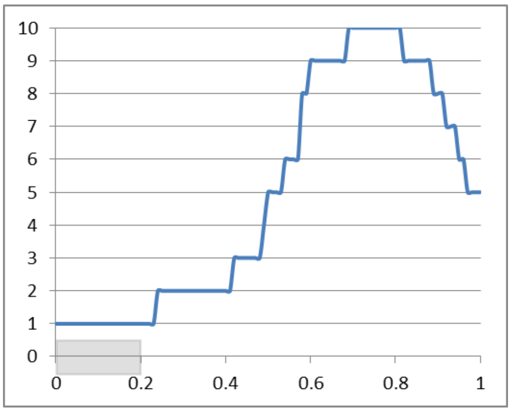

3) How likely is it that *Banksia attenuata* is susceptible to *Phytophthora cinnamomi* dieback?

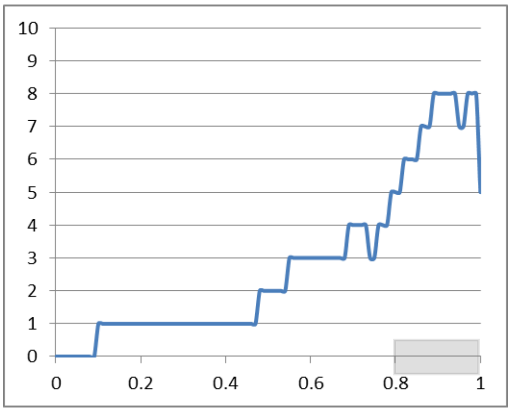

4) Compared to the other vegetation elements, how likely is it that the natural species richness of the Mallee shrubland vegetation community is the highest?

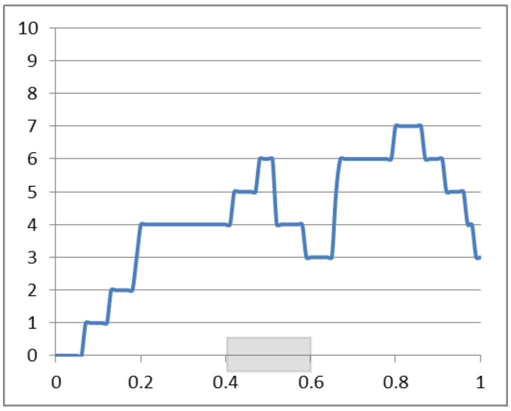

5) With regards to grazing pressure on the natural plants of the Mallee shrubland element, how likely is it that a density of 3 rabbits per 10 ha would be considered a high density?

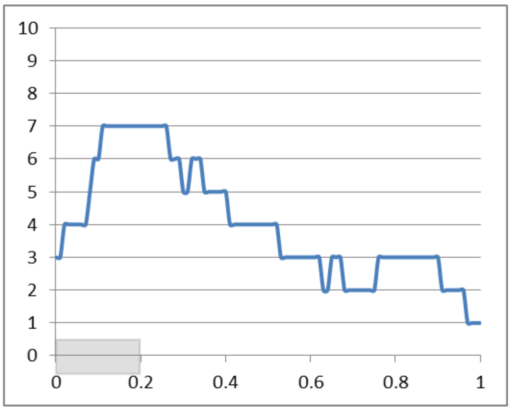

6) How likely is it that the presence of highly saline ground water (>50,000 mg/L) within 1 meter of the surface of Lake Bryde for 12 months will cause a high degree of mortality in *Duma horrida*?

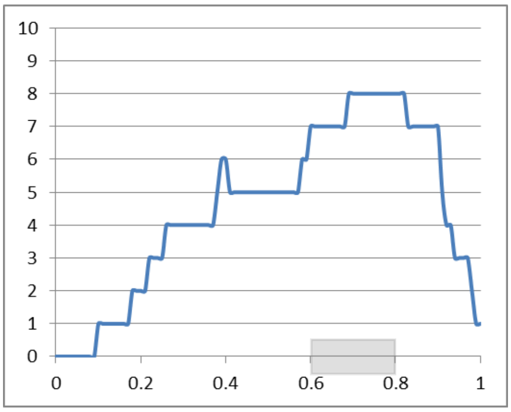

7) How likely is it that fox and cat predation on eggs is a more significant risk factor for Mallee Fowl than fox and cat predation on chicks?

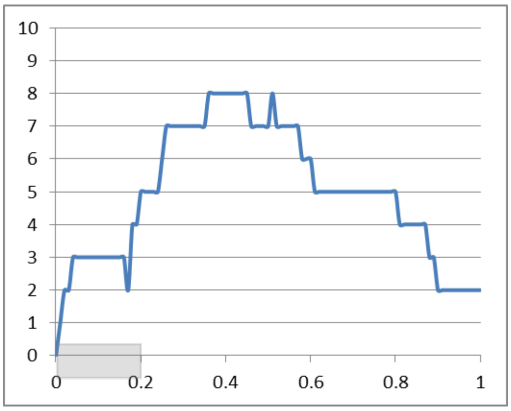

8) How likely is it that interactions between fire regime and vegetation response will have a strong influence on skink diversity?

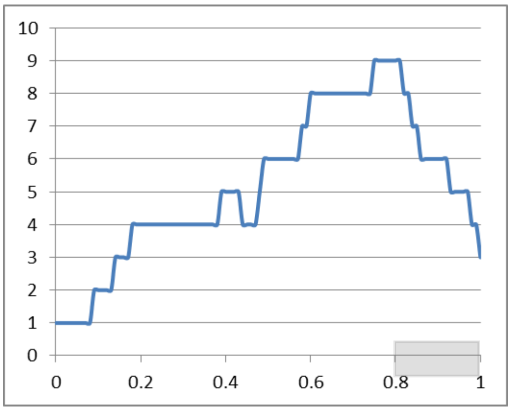

9) How likely is it that *Eucalyptus occidentalis* can resprout from a lignotuber?

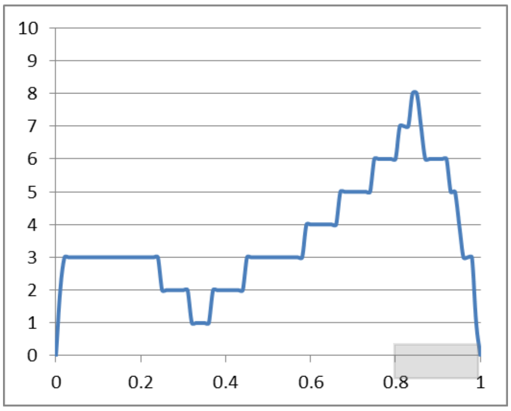

Scoring the expert calibration questions.

To calibrate each expert, the proportion of their interval that overlapped with the correct likelihood for each calibration question was calculated (Figure S1.2). The expert's average proportion (across all 9 questions) was used as their overall calibration score.

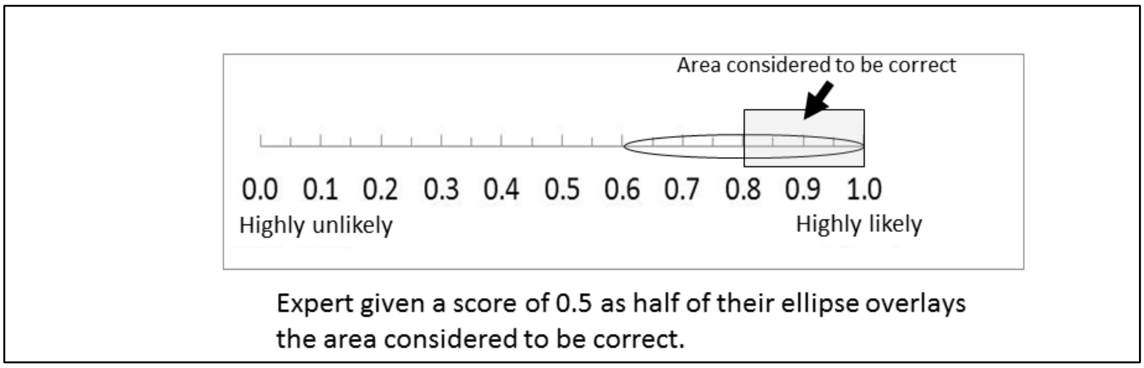

Figure S1.2: Description of the approach used to score each expert's calibration answers.
