# Supplementary material for: A structured elicitation method to identify key direct risk factors for the management of natural resources
Source: Heliyon. 2015 Nov 24;1(3):e00043. doi: 10.1016/j.heliyon.2015.e00043 (PMC4945618; doi:10.1016/j.heliyon.2015.e00043)
Supplement: Supplementary material 3 [file mmc3.pdf]

Supplementary Material 3: Expert raw-, CI-, Calibrated-estimates and the aggregation results and some examples of aggregation graphs for a selection of risk factor-element combinations.

Table S3.1: Estimated likelihood of causing management target failure (loss of natural species) for each important (initially classed by the expert group as >0.05) risk factor-element combination. Raw min-max estimates, CI-estimates and Calibrated-estimates are reported. Diff — the difference between the Calibrated-estimate and the Raw-estimate — provides an indication of the degree of influence the better calibrated experts had on the estimates. The largest difference scores (≥ 0.1) are marked by an asterisk. Data are ordered by the Calibrated-estimates.

**Risk**	**Element**	Raw	CI	Cal	Diff
Salinity*	Aquatic Invertebrates	0.20	0.20	0.44	0.24
Lack of Water (Drought) *	Other Woodlands	0.20	0.39	0.39	0.19
Lack of Water (Drought)	Amphibians	0.30	0.29	0.29	-0.01
Lack of Water (Drought) *	*Melaleuca* Shrubland	0.17	0.29	0.29	0.12
Food*	Aquatic Invertebrates	0.10	0.27	0.27	0.17
Salinity	Other Woodlands	0.25	0.25	0.25	0.00
Salinity	Salmon Gum	0.20	0.20	0.20	0.00
Salinity*	Yates Swamp	0.10	0.20	0.20	0.10
Lack of Water (Drought)	Mallee Shrubland	0.20	0.19	0.19	-0.01
Salinity	*Duma horrida* vegetation community	0.20	0.19	0.19	-0.01
Food	Waterbirds	0.20	0.19	0.19	-0.01

Oxygen (water logging)	Mallee Shrubland	0.19	0.18	0.18	-0.01
Salinity	*Melaleuca* Shrubland	0.20	0.18	0.18	-0.02
Lack of Water (Drought)	Waterbirds	0.18	0.16	0.16	-0.02
Salinity	Amphibians	0.15	0.15	0.15	0.00
Predation	Mammals	0.15	0.15	0.15	0.00
Temperature*	Terrestrial Birds	0.05	0.15	0.15	0.10
Lack of Water (inappropriate hydroperiod)	Aquatic Invertebrates	0.10	0.12	0.12	0.02
Physical Damage (including fire)	Mallee Shrubland	0.11	0.11	0.11	0.00
Oxygen (water logging)	Other Woodlands	0.20	0.11	0.11	-0.09
Acidity/Alkalinity	Aquatic Invertebrates	0.10	0.10	0.10	0.00
Oxygen (water logging)	*Melaleuca* Shrubland	0.10	0.10	0.10	0.00
Physical Damage (including fire)	*Melaleuca* Shrubland	0.10	0.10	0.10	0.00
Salinity	Waterbirds	0.10	0.10	0.10	0.00
Oxygen (water logging)	Yates Swamp	0.10	0.10	0.10	0.00
Temperature	Mammals	0.05	0.09	0.09	0.04
Pesticides/Herbicides	Terrestrial Invertebrates	0.00	0.09	0.09	0.09
Disease	*Melaleuca* Shrubland	0.10	0.08	0.08	-0.02
Physical Damage (including fire)	Salmon Gum	0.10	0.08	0.08	-0.02
Grazing	Yates Swamp	0.10	0.08	0.08	-0.02
Disease	Mallee Shrubland	0.05	0.07	0.07	0.02

Physical Damage (including fire)	Other Woodlands	0.05	0.06	0.06	0.01
Oxygen (water logging)	Salmon Gum	0.10	0.06	0.06	-0.04
Predation	Terrestrial Birds	0.09	0.06	0.06	-0.03
Salinity	Mallee Shrubland	0.05	0.05	0.05	0.00
Salinity	Samphire	0.05	0.05	0.05	0.00
Acidity/Alkalinity	Amphibians	0.04	0.04	0.04	0.00
Grazing	*Melaleuca* Shrubland	0.10	0.04	0.04	-0.06
Grazing	*Duma horrida* vegetation community	0.01	0.04	0.04	0.03
Disease	Salmon Gum	0.05	0.04	0.04	-0.01
Grazing	Samphire	0.00	0.04	0.04	0.04
Pesticides/Herbicides	Terrestrial Birds	0.05	0.04	0.04	-0.01
Physical Damage (including fire)	Yates Swamp	0.05	0.04	0.04	-0.01
Lack of Mates	*Melaleuca* Shrubland	0.05	0.03	0.03	-0.02
Disease	Yates Swamp	0.05	0.03	0.03	-0.02
Grazing	Mallee Shrubland	0.05	0.02	0.02	-0.03
Grazing	Salmon Gum	0.10	0.02	0.02	-0.08
Pesticides/Herbicides	Waterbirds	0.05	0.02	0.02	-0.03
Pesticides/Herbicides	Amphibians	0.05	0.01	0.01	-0.04
Lack of Mates	Mallee Shrubland	0.00	0.01	0.01	0.01
Disease	*Duma horrida* vegetation community	0.00	0.00	0.00	0.00
Grazing	Other Woodlands	0.00	0.00	0.00	0.00

Acidity/Alkalinity	Yates Swamp	0.00	0.00	0.00	0.00

Selected examples of aggregation graphs for a subset of risk factor-element combination

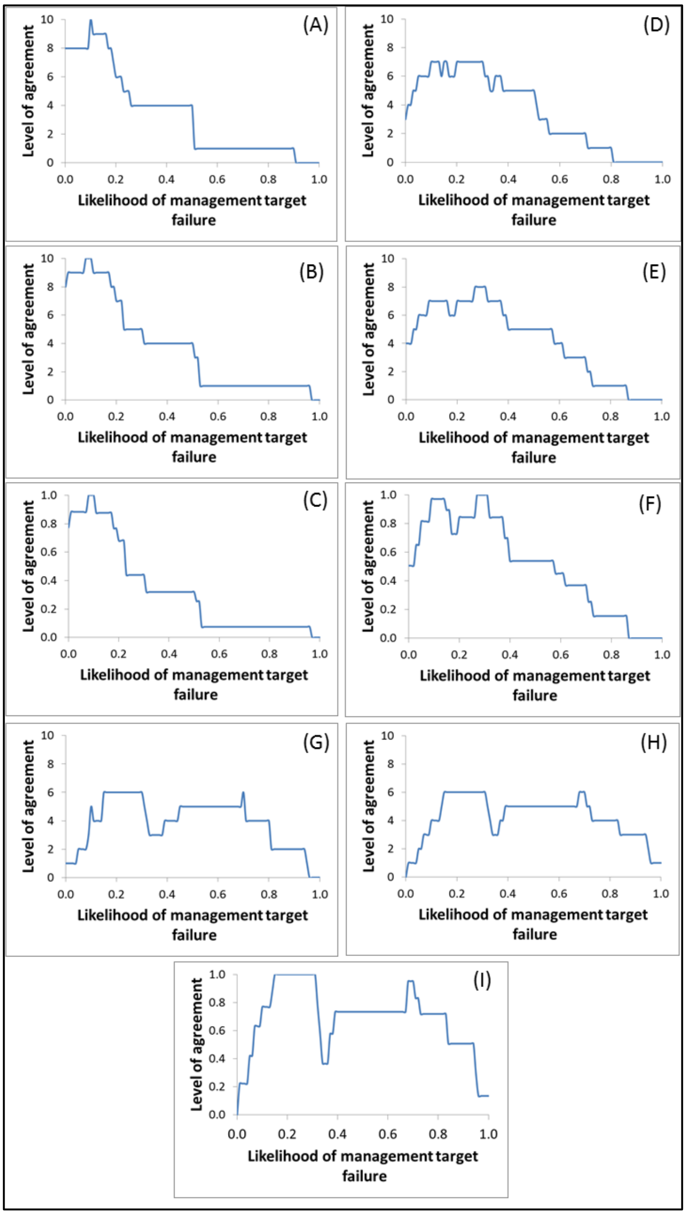

Fig. S3.1: Disease-*Melaleuca* shrubland showing raw expert data (A), CI-adjusted data (B) and Calibrated-estimates (C). Food (starvation)-aquatic invertebrates showing raw expert data (D), CI-adjusted data (E) and Calibrated-estimates (F). Predation-mammals showing raw expert data (G), CI-adjusted data (H) and Calibrated-estimates (I). Calibrated-estimates have been normalised to vary between 0 and 1. The x-axis represents the likelihood of causing management target failure and the y-axis represents the level of agreement across the expert-group.
